# Recruitment, Training, and Roles of the Bilingual, Bicultural *Navegantes*: Developing a Specialized Workforce of Community Health Workers to Serve a Low-Income, Spanish-Speaking Population in Rhode Island

**DOI:** 10.3389/fpubh.2021.666566

**Published:** 2021-06-04

**Authors:** Meghan McCarthy, Katherine Barry, Cindy Estrada, Brenda Veliz, Damaris Rosales, Morgan Leonard, Anne S. De Groot

**Affiliations:** ^1^Clínica Esperanza/Hope Clinic, Providence, RI, United States; ^2^The Warren Alpert Medical School of Brown University, Providence, RI, United States; ^3^EpiVax, Inc., Providence, RI, United States; ^4^University of Georgia, Athens, GA, United States

**Keywords:** community health worker, community-based job training, health promotion, patient navigation, medical interpretation, Spanish-speaking communities of the US, public health workforce

## Abstract

Clínica Esperanza/Hope Clinic (CEHC) employs *Navegantes*, who are specially-trained bilingual Community Health Workers (CHW), as key team members who improve the ability of the clinic to provide care for and improve the health status of a large population of uninsured Spanish-speaking patients in Providence, Rhode Island. Given the growing demand for CHWs at the clinic and in the broader healthcare sector in the state, CEHC developed the Advanced *Navegante* Training Program (ANTP). The ANTP prepares community members to become certified CHWs who are equipped to provide patient navigation and lifestyle coaching as well as professional medical interpretation services. The ANTP is developed and taught by CEHC *Navegantes* who themselves are bilingual and bicultural peers of trainees as well as the population that CEHC serves. Upon graduation, ANTP trainees have been able to attain higher-paying and fulfilling careers in a range of healthcare and other community settings. The ANTP offers a low-cost, community-based model for training CHWs who are uniquely prepared to promote health and well-being among medically underserved patients.

## Introduction

Community health workers (CHW) are in high demand in the healthcare and non-profit industries. The US Department of Labor recently projected that health educator and community health jobs will increase by 16% from 2016 to 2026 (1). In Rhode Island, growth in this job sector is currently driven by state-level efforts to improve access to healthcare and health information in underserved communities. Engagement of CHWs is also viewed as a means of reducing healthcare costs by teaching people healthy behaviors and connecting them to healthcare services, which may help to reduce health disparities (2).

Bilingual CHWs are particularly sought after to serve as healthcare navigators, patient advocates and medical interpreters in settings where there is a substantial Spanish-speaking patient population. In this capacity, bilingual and bicultural CHWs can serve as a linguistic and cultural bridge between clinicians and their patients, assisting with medical interpretation, patient education, adherence monitoring, and connections to social services (3).

A recent assessment of the CHW employment sector in Rhode Island reported that Community Health Workers work in a diverse set of medical settings including Federally Qualified Health Centers, hospitals, health systems, clinics and insurance companies. Many CHWs also work in community-based organizations that combine social and health services, such as Community Action Programs (CAPs), education systems, and local Health Equity Zones (4).

## Context

Clínica Esperanza/Hope Clinic (CEHC) is a free healthcare clinic in West Providence, Rhode Island that serves a population of over 3,000 uninsured, low-income Spanish-speaking immigrants. Recognizing that patients would benefit from the guidance of bilingual and bicultural healthcare navigators and community health workers, CEHC instituted this role at the time of its founding in 2007. At that time, the clinic was entirely run by volunteers and operated out of a church basement. Community members who took on the role of *Navegantes* were trained to help with measuring blood pressure, checking basic laboratories such as blood sugar and cholesterol, and connected the clinic volunteers to members of communities that were hard hit during the 2008 recession.

The *Navegantes* continued to play a major role in the operation of CEHC as its services grew and it moved to a new, permanent location in 2010. Their participation in local health fairs attracted new uninsured patients to the clinic, as *Navegantes* helped foster trust and visibility of CEHC services in the community. Over the past 10 years, the *Navegantes* have engaged in a wide variety of health-promoting activities in the community, such as conducting community health needs assessments and door-to-door Census education, participating in active outreach and special health education programs at local community centers, and representing CEHC at city, state, and regional meetings.

The *Navegantes* also continue to fulfill multiple roles in the daily clinical operations of CEHC. CEHC is staffed with a team of *Navegantes* (typically 3–4) during all operating clinical hours to provide patient navigation, social service referrals, and peer-to-peer healthy lifestyle education. One *Navegante* each day is assigned to provide medical interpretation services. The *Navegantes* are also responsible for providing group session on healthy lifestyles including the CEHC's own Vida Sana lifestyle program and the Diabetes Prevention Program (DPP), funded by the Rhode Island Department of Health (RI DOH) (5, 6).

In 2012, as opportunities for bilingual and bicultural CHWs in RI continued to expand, CEHC recognized the opportunity to formally train and hire new *Navegantes* and developed the Advanced *Navegante* Training Program (ANTP). The goal of the program is to expand the number of formally trained and certified bilingual and bicultural CHWs that are particularly well-equipped to care for the Spanish-speaking immigrant populations. From the advent of this program, the curricular and logistical planning has been spearheaded by staff *Navegantes*, with the support of project managers and administrators at CEHC.

In addition to developing a workforce to meeting critical community health needs, the ANTP is also designed to empower individuals with the skills needed to attain rewarding and higher paying jobs. The ANTP has been organized and taught by current *Navegantes* who know best how to serve this community. The goal of the ANTP is to build a workforce that will empower CHW to become advocates for improving access to healthcare for their community. Here, we describe practical components and lessons learned from developing and offering a community-based CHW training program at a volunteer-run free clinic.

## Key Programmatic Elements

### Recruitment

CEHC recruits and trains one to two cohorts of 12–15 participants in the ANTP per year. Each year before the ANTP begins, information is distributed to community members through partner organizations and social media posts. In collaboration with community partners, CEHC acts as the central recruitment agency and identifies potential participants through a variety of methods, including emailing listservs connected to healthcare job seekers, posting flyers at job fairs and outreach events, sponsoring ads on Facebook, and notifying our patient population about the training opportunity.

Participants are also recruited by word of mouth through prior *Navegante* graduates who spread awareness about the opportunity within their social networks. Many of these individuals have obtained jobs in local healthcare offices and community-based organizations, so they have made connections with other individuals who are interested in advancing their careers. Since the focus of the ANTP is to train members of the community who can provide culturally appropriate care to patients with similar life experiences, using local networks to recruit participants has proven to be an effective method of recruitment.

### Selection Processes

Individuals interested in the training are asked to send a resume and a letter of interest that describes their reason for wishing to participate in the course. Applicants to the ANTP are screened by the CEHC *Navegantes* and offered an interview if they meet the following criteria: (1) can commit to the time that is required to complete the course, (2) are bilingual in English and another language (Spanish or Portuguese have historically been prioritized given CEHC's patient population, but participants who speak other languages such as Creole, North African, or Middle Eastern languages spoken by refugees have enrolled), and (3) have at least a high school diploma or GED, classroom experience and/or several years of work experience.

We aim to include participants in the program who are interested in seeking employment in (or currently employed in) a healthcare setting, but do not currently have the knowledge and/or skills to carry out their desired roles. Usually there are more interested candidates than space in the program allows, resulting in a waiting list almost as long as the number enrolled. In these cases, the selection process is based on the applicant's interest in and dedication to the training, their relevant qualities skills (i.e., language skills, inclination for collaborative teamwork, etc.) and their demonstrated ability to perform well in an intensive classroom setting.

### Training Components

The Advanced Navegante Training program (ANTP) is an intensive 10-weeks didactic program followed by an experiential learning internship. Classes are held during evenings and on weekends to accommodate participants' work schedules. Classroom learning takes place during 1–2-h sessions on weeknights, and 3–4-h sessions on the weekends. It is supplemented by at least 80 h of clinic-based experiential learning, where ANTP trainees can work with their mentors (the current *Navegantes*), assisting patients and interpreting for clinicians. A list of the topics and skills covered in the ANTP is provided in Table 1.

**Table 1:** ANTP curricular components.

Certifiable Health Care Navigator Training (using CDC workbooks) • Sessions on case management, role of CHWs • Basic Clinical Practice, including HIPAA training and medical ethics • On-site one practicum, one evening per week for 10 weeks
Chronic disease short course • Diabetes, hypertension, Cardiovascular Disease (visiting lectures) • Instructors include: CEHC providers, including MD, PA, RNP, medical students
Case management training
First AID/CPR training
Formal medical interpreter training course • Open to former ANTP graduates and other CHW training program graduates
Additional health topics/sessions • Domestic violence • Cancer prevention and screening • Women's health • Sexual health/gender identity • COVID-19 and vaccination
Lifestyle change classes • Vida Sana • Diabetes Prevention Program (two 8-hr sessions provided by RI DOH)
Human subjects research studies certification • Provided by CEHC with online modules from the Collaborative Institutional Training Initiative

A key aspect of the ANTP is that it is peer-taught by current CEHC *Navegantes*, who create a comfortable and supportive environment. The experienced *Navegantes* are familiar with the personal backgrounds and experiences of the participants, improving the pedagogical efficacy and practical implementation of the program for both trainers and trainees.

The ANTP uses CDC-approved, evidence-based course materials as the backbone of the Health Care Navigator training course. Specifically, the clinic uses “A Community Health Worker Training Resource for Preventing Heart Disease and Stroke,” which is an online manual available on the CDC website (7). The downloadable resource is printed for each of the participants in the course. Written in plain English, the CDC resource has 15 chapters on topics such heart disease, stroke, high blood pressure and cholesterol, diabetes, depression and stress, medication adherence, and other lifestyle risk factors.

During the classroom-based learning portion of the program, the CDC CHW curriculum is supplemented with other resources and guest speakers, which allows ANTP participants to gain knowledge in a wide variety of extra topics, such as chronic disease prevention and management, case management, and professional boundaries. This also ensures that the ANTP curriculum covers the key domains of knowledge outlined by the RI DOH (8). *Navegante* course leaders invite CEHC staff, volunteers, and guest speakers from the community to share their expertise on these topics. Visiting medical and nursing faculty from Brown University and University of Rhode Island come to instruct sessions on specific topics that are required for CHW certification, and honoraria for these speakers are included in the ANTP budget.

The trainees benefit from hearing the real-life context that the *Navegantes* and expert guest speakers offer related to the course subject matter as it provides them information about a wide range of healthcare settings. In addition, by telling stories about their own experiences, these speakers enable CHW-trainees to learn first-hand about what future employment opportunities they might be interested in pursuing.

### Medical Interpretation

The Medical Interpreting course is provided over 8 weeks of intensive classroom training (6 h per weekend) by a certified medical interpretation educator. Participants learn about the health care interpreting profession and receive formal training in language and communication, professional ethics, health care systems, culture, medical interpreting protocols, message conversion, modes of interpretation, cultural brokering, mental health, HIPAA, job readiness (resume, cover letter, reference, and job searching strategies), professionalism, and customer service. The course also reviews basic medical conditions and medical terminology that interpreters may encounter on the job.

### Lifestyle Class Leadership Training

A major role for CHWs is to provide health coaching and lead classes on healthy lifestyles. ANTP participants receive training in two types of lifestyle classes. The first is the Vida Sana/Healthy Life program, which is a unique social group-based course that was created and implemented by CEHC staff (5, 9). Vida Sana is structured as an interactive 8-weeks course that teaches participants basics about nutrition, making healthy choices, and self-management of chronic diseases for individuals with low health literacy. As part of ANTP, trainees are required to learn to lead Vida Sana classes by way of a “see one, do one, teach one” model. As part of the classroom component of the training, they take a Vida Sana class as participants, then help to teach one under the mentorship of experienced staff CEHC *Navegantes*. In this way, the *Navegantes* can experience the impact of healthy lifestyle training themselves, helping them learn the techniques to be effective at teaching it to others. In addition, the material covered in Vida Sana reinforces information presented to trainees during the didactic portion of the program. This iterative training process prepares participants to successfully conduct health coaching and facilitate the Vida Sana class to future cohorts. Upon graduation, participants receive a certificate of completion to facilitate the Vida Sana/Healthy Life program as a Lifestyle Coach at CEHC.

The Diabetes Prevention Program (DPP) is an evidence-based lifestyle change program through the CDC. The DPP Lifestyle Coach training is provided to ANTP participants by the RI DOH. In the DPP, trained lifestyle coaches lead group classes to help participants at risk for diabetes to improve their food choices, increase physical activity, and learn coping skills to maintain weight loss and healthy lifestyle changes to prevent the development of diabetes (6).

Upon completion of ANTP, participants receive a certification to facilitate both the DPP and Vida Sana as a *Navegante*, qualifying these individuals for employment opportunities at local clinics and hospitals, and in programs such as the Health Equity Zone programs funded by the RI DOH.

### Additional Components

In addition to the CHW certification they earn, participants in ANTP graduate with other marketable skills that serve the needs of the employers who are in need of highly trained patient advocates. These include First Aid/CPR Certification and Human Subjects Research Training through the Collaborative Institutional Training Initiative website.

### Evaluation

Participants in the ANTP are required to complete and regular exams in order to progress through the course. The CHW training component includes four quizzes and one final exam, while the medical interpreter training includes a pre-test, midterm, and final exam. Other components, including Vida Sana, include quizzes to assess participant understanding and readiness to continue.

### Experiential Learning Component

As part of ANTP, participants are required to complete an additional 80 h of on-site training at CEHC, for which participants receive a stipend (funded through the RI Department of Labor and Training). Since the majority of the participants either work full-time or work more than one job, these hours are very flexible (nights, weekends), and they have an entire year after graduation from the didactic phase to complete the additional 80 h.

During this experiential learning component, ANTP trainees shadow the staff *Navegante*s, who have a unique and broad set of duties and roles at CEHC. *Navegante* duties include administrative/clerical work, social service and healthcare navigation, medical interpreting, facilitating lifestyle classes, and more. If the ANTP trainees are also certified Medical Assistants (as many are), they can also perform Medical Assistant duties during CEHC clinics, such as taking vitals, administering point of care testing, urinalysis, vaccines, etc.

In some cases, ANTP trainees are offered employment at organizations outside CEHC before completing the experiential component of their training. In these cases, trainees are able to receive state CHW certification after completing the requisite number of hours at their new job.

### Graduation and CHW Certification

Upon completing each of the requirements and passing each of the assessments, the participants are eligible to receive official certifications as a RI DOH Community Health Worker, a Professional Medical Interpreter, a DPP and Vida Sana Lifestyle Coach, and a First Aid/CPR provider.

In Rhode Island, participants can receive formal CHW certification upon successful completion of both the classroom portion of ANTP, as well as 1,000 h and/or 6 months of work as a CHW (8). Trainees also have the opportunity to become certified professional Medical Interpreters (predicated on their completion of the Medical Interpreter oral and written exam).

### Costs and Funding

The ANTP is provided free of cost to participants. For the first several years, ANTP was entirely supported by grant funding from local philanthropic organizations, including the Textron Foundation. In 2019, CEHC provided a partnership with the RI Department of Labor and Training (DLT) which established a renewable mechanism for funding for ANTP. DLT funding has allowed CEHC to expand the program from one to two cohorts per year (graduating about 30 participants annually) as well as providing additional funding for direct participant stipends.

The total cost to train each participant is about $7,500, which includes the cost of the RI CHW certificate ($125), the National Medical Interpreter Exam ($450), medical equipment ($125), and CPR certification ($75), and a stipend for the experiential learning component ($500). In addition to these direct costs that are covered for participants, they receive one-on-one mentoring with a senior *Navegante* during their internship, assistance with career placement, and networking opportunities. The cost per participant also includes operational expenses associated with running the program like honoraria for presenters from The Warren Alpert Medical School of Brown University and local hospital systems including Lifespan and Care New England, as well as salaries for the *Navegante* leaders.

### Strategic Partners

ANTP is based on a strong community collaboration of organizations that provide various components of the training, spread the word for recruitment, facilitate job placement after graduation, and more. These organizations span many different industries, from small non-profits, social service agencies, universities, state agencies, to large healthcare systems.

## Discussion

ANTP is offered on site at a free clinic that serves uninsured patients, many of whom are Spanish-speaking immigrants. In addition to providing valuable job training and improving employment outcomes for participants, the ANTP responds to the critical need for culturally competent and linguistically appropriate health care for medically underserved members of the Rhode Island community.

### Outcomes

Since 2012, CEHC has trained over 100 bilingual and bicultural community members in the ANTP (Table 2). Of ANTP graduates who sought employment following the program, many have found rewarding and high-paying jobs in a variety of fields. Through CEHC's established partnership network with many institutions that employ CHWs or medical interpreters, ANTP graduates have been hired to work in health clinics, community-based organizations, and hospitals. For example, several graduates are working as full-time healthcare interpreters in hospital settings and emergency rooms. Others have supplemented their family incomes by becoming part time medical interpreters during evening hours. Other graduates have secured jobs with benefits as outreach workers in local health systems and social service agencies such as Providence Community Health Centers, Family Services RI and Neighborhood Health Plan. CEHC is also able to hire several new *Navegantes* from each of the graduating classes to work at CEHC depending on funding and staffing needs.

**Table 2:** ANTP outcomes by year.

**Year**	**# ANTP graduates**	**# Employed at CEHC**
2015	12	4
2016	10	2
2017	15	1
2018	16	2
2019^*^	31	4
2020*^†^	25	2

* *Starting in 2019, we received funding to conduct 2 cohorts per year.*

† *All 2020 cohorts participated in the virtual ANTP format due to the COVID-19 pandemic.*

*This table provides information on the number graduates from the didactic portion of ANTP. A small number go directly into full-time employment following graduation, while most complete the experiential learning internship (80-h internship at CEHC)*.

Through this training, low- to moderate-income level participants gain access to more rewarding and higher paying jobs in the growing sector of employers hiring CHWs and patient advocates in the healthcare industry. On average, CHWs earn $21.91 per hour in Rhode Island, more than the minimum wage in Rhode Island ($11.50 per hour) (10). With this extensive training, participants can advance their careers and become future leaders in the field of community health, often moving into higher-level management positions in many social service and healthcare agencies.

Most graduates are now certified CHWs and engaging in fulfilling, higher-paying employment at CEHC and other community-based organizations and healthcare systems. These cohorts of ANTP graduates have been empowered and equipped with the tools to help community members overcome the many cultural and linguistic barriers that arise within our healthcare system, leading to long lasting, positive effects on the health of all members of the community in Rhode Island.

### Testimonials

During the ANTP graduation, participants often share powerful testimonials of their experience with this program and their gratitude for the opportunity. Some excerpts can be found in Table 3. Most were offered in Spanish, but the translated version is provided here.

**Table 3:** Testimonials from ANTP graduates.

“Not only I was able to learn about community resources and what makes a good community health worker; I was able to learn from each and every one of my classmates, presenters, and *Navegantes*. I treasure the time spent with them and the contributions they have made to my life.”
“This course has opened up my eyes to a vital, often overlooked need in the healthcare system. I have always been someone who naturally wants to help. Being a proud resident of Providence, born and raised, it feels amazing to know that this knowledge gained will allow me to be part of empowering my community and provide support in many aspects. Thank you to all the presenters who took time out of their busy days to come teach our tired minds. It was appreciated how you all kept the information interesting and engaging. For all the silly games that kept our blood rushing and all the laughs. For answering all of our questions and providing visuals of how much sugar was in many of our favorite drinks. And for all the amazing life hacks/tips/tricks that I will utilize in my personal life as well as professionally. I am forever grateful.”
“Many of us, our parents or grandparents, arrived in this country as immigrants; some maybe fleeing from violence, wars, poverty in our countries. While they taught us how to help people, families and the community in many of the social and socioeconomic problems we face, we also learned how to prevent diseases, and have a healthy lifestyle through the Healthy Life program, and we learned a third language as Medical Interpreters, that is Medical Terminology to also be able to help people who need a translator in their medical appointments.”

*Most of these testimonials were offered in Spanish, and the translated version is provided here*.

### Strengths of the Program

A key to success for this program has been ANTP's peer-led model. The advantages of this model are two-fold: (1) the *Navegantes* are peers of the current trainees and have shared personal and professional experiences, improving the pedagogical and practical efficacy of the program, and (2) in providing the training themselves, the *Navegantes* receive ongoing education in critical topics, becoming more experienced and skilled CHWs in the process. They create an environment for the training that is welcoming and supportive while ensuring that participants acquire skills that they believe to be essential to be successful as CHW.

In addition, the ANTP curriculum is designed to be multifaceted so that each participant involved gains the maximum benefits and are prepared for a variety of employment settings. ANTP training provides a number of tangible skills and certifications, from health coaching and case management to medical interpretation. The diverse set of skills they obtain make ANTP graduates marketable to a wide variety of future employment settings.

## Conceptual and Methodological Limitations

### Challenges and Lessons Learned

An important focus of ANTP is that its trainees come from the same economically and medically underserved population that they intend to serve. This is important because it provides opportunities for people with limited educational background to seek higher paying, fulfilling jobs as CHWs and community advocates. However, during early cohorts of ANTP, we found that some participants faced significant barriers to completing the standardized assessments that are required to gain the formal certifications as CHWs or medical interpreters. As the demand for the program increased significantly, more careful screening procedures were instituted during recruitment which have helped to identify applicants who may require additional support to succeed during those parts of the program. Individuals who are not selected upon their first application to the ANTP are offered opportunities to volunteer at CEHC or other resources for job training that may prepare them for the program in the future.

Another significant challenge has been finding a steady source of comprehensive funding. During the early years of this program, CEHC only had a limited budget to fund the operational costs of the program. For some ANTP trainees, the costs of certification (for the state medical interpreter or CHW certification) and the unfunded requirement for additional on-site training was a significant barrier to success. However, CEHC's recent partnership with the RI DLT has allowed for a significantly larger budget for these additional components of the training, covering both these certification costs and stipends for the experiential learning components as well as providing continuity in funding for CEHC as the parent organization.

### Online Adaptation and COVID-19

In early spring 2020, the ANTP was moved to a virtual format due to the COVID-19 pandemic. There have been some significant challenges associated with transferring this program online. For example, some trainees lacked the necessary technology (such as a computer and reliable WiFi connection) to participate in the online classes, but CEHC staff were able to work with trainees to find other options (e.g., phones, tablets) to ensure their ability to participate. In addition, the nature of the ANTP typically lends itself to a very dynamic classroom atmosphere with interactive activities and discussions. The comradery among trainees that naturally develops among the trainees in the classroom has also been more difficult to achieve in a virtual format. The *Navegante* course leaders have also noted that it is also more difficult to recreate this atmosphere and keep the attention of the participants in a virtual format. However, the online format did allow for two cohorts of ANTP trainees to successfully graduate despite the COVID-19 pandemic. It also allowed more flexibility for those who may not have otherwise been able to participate, including trainees who worked in evening hours and those with children.

### Implementation Strategies and Next Steps

One central implementation strategy to the ANTP is its integration within the greater parent organization of CEHC. Designing this program under the umbrella of CEHC's work as a free clinic provides the opportunity to leverage the existing infrastructure and staffing, which helps to minimize overhead and operational costs. Importantly, one of the major roles of full-time CHWs at CEHC is to coordinate and lead the entire ANTP with the support of the clinic's existing resources. It also allows graduates to benefit from the existing partnership network of CEHC for job placement. An additional advantage of this model is that CEHC is able to fulfill staffing needs by hiring several new ANTP participants for part- or full-time positions upon graduation.

Another important aspect of the ANTP is making the work of CHWs at the center of all components of the program. The peer-led model enhances the warm social atmosphere and community among participants that is developed throughout the program. In this way, the program remains true to its core values of bridging the cultural and language gap that often exists within our healthcare and social service systems.

An important next step of this program is to scale up and adapt the program for other community organizations within Rhode Island and beyond. Given the flexibility of its design, the ANTP is thought to be highly adaptable to other settings and could be recreated in many places around the country.

## Conclusions

CHWs are in high demand in healthcare and non-profit industries. ANTP is a relatively low-cost, sustainable and community-based job training model that uniquely prepares graduates to work in a variety of settings. By providing them with the tools they need to become CHWs, participants have access to higher-paying and fulfilling jobs, as we also build a growing team of knowledgeable, passionate advocates who promote community health for the medically underserved.

## Data Availability Statement

The original contributions presented in the study are included in the article/supplementary material, further inquiries can be directed to the corresponding author/s.

## Author Contributions

MM and KB wrote the first draft of the manuscript. CE, BV, and DR were involved in the initial development and implementation of the ANTP, provided the logistical information, and details that were used to write the manuscript. ML and ADG oversaw the program's implementation and wrote sections of the manuscript. All authors helped to revise, read, and approved the submitted version of the manuscript.

## Conflict of Interest

ADG was employed by the company EpiVax, Inc., which was not involved in this work financially or otherwise. The remaining authors declare that the research was conducted in the absence of any commercial or financial relationships that could be construed as a potential conflict of interest.

## References

[1] Health Educators Community Health Workers: Occupational Outlook Handbook. U.S. Bureau of Labor Statistics. (2019). Available from: https://www.bls.gov/ooh/community-and-social-service/health-educators.htm (accessed January 17, 2021).

[2] Alexander-Scott N Garneau D Dunklee B. Community Health Workers in Rhode Island: Growing a Public Health Workforce for a Healthier State. Providence, RI: Department of Health (2018)

[3] Balcazar H Lee Rosenthal E Nell Brownstein J Rush CH Matos S Hernandez L. Community health workers can be a public health force for change in the United States: Three actions for a new paradigm. Am J Public Health. (2011) 101:2199–203. 10.2105/AJPH.2011.300386 22021280 PMC3222447

[4] Dunklee B Garneau D. Community health workers in rhode island: a study of a growing public health workforce. R I Med J. (2013) 101:40–3. 30068054

[5] Buckley J Yekta S Joseph V Johnson H Oliverio S De Groot AS. Vida Sana: a lifestyle intervention for uninsured, predominantly spanish-speaking immigrants improves metabolic syndrome indicators. J Community Health. (2014) 40:116–23. 10.1007/s10900-014-9905-z 24984599

[6] Group TDPP (DPP) R. The Diabetes Prevention Program (DPP): description of lifestyle intervention. Diabetes Care. (2002) 25:2165–71. 10.2337/diacare.25.12.2165 12453955 PMC1282458

[7] A Community Health Worker Training for Preventing Heart Disease and Stroke. Available online at: https://www.cdc.gov/dhdsp/programs/spha/chw_training/pdfs/chw_training.pdf (accessed January 17, 2021).

[8] Community Health Workers: Department of Health. Available from: https://health.ri.gov/communities/about/workers/ (accessed January 17, 2021).

[9] Risica PM McCarthy M Barry K Oliverio SP De Groot AS. Community clinic-based lifestyle change for prevention of metabolic syndrome: rationale, design and methods of the ‘Vida Sana/healthy life' program. Contemp Clin Trials Commun. (2018) 12:123–8. 10.1016/j.conctc.2018.10.002 30417157 PMC6218840

[10] Rhode Island Occupational Employment and Wage Estimates. Bureau of Labor and Statistics (2019). Available online at: https://www.bls.gov/oes/current/oes_ri.htm (accessed January 17, 2021).

